# Terapia Trombolítica em Octogenários com Embolia Pulmonar Aguda

**DOI:** 10.36660/abc.20201060

**Published:** 2021-11-17

**Authors:** Ahmet Zengin, Mehmet Baran Karataş, Yiğit Çanga, Özge Güzelburç, Nizamettin Selçuk Yelgeç, Ayşe Emre

**Affiliations:** 1 Department of Cardiology University of Health Sciences Dr. Siyami Ersek Training and Research Hospital Istambul Turkey Department of Cardiology , University of Health Sciences , Dr. Siyami Ersek Training and Research Hospital , Istambul - Turkey

**Keywords:** Octogenários, Prognóstico, Fatores de Risco, Terapia Trombolítica, Mortalidade, Hemorragia/complicações

## Abstract

**Fundamento:**

Apesar da grande proporção de octogenários com embolia pulmonar aguda, há pouca informação indicando a estratégia de manejo ideal, especialmente medidas terapêuticas, como a terapia lítica.

**Objetivos:**

O número de pacientes idosos diagnosticados com embolia pulmonar aguda aumenta constantemente. Porém, o papel do tratamento trombolítico não está claramente definido entre os octogenários. Nosso objetivo é avaliar a efetividade da terapia lítica em pacientes octogenários diagnosticados com embolia pulmonar.

**Métodos:**

Cento e quarenta e oito indivíduos (70,3% de mulheres, n=104) com mais de 80 anos foram incluídos no estudo. Os pacientes foram divididos em dois grupos: tratamento trombolítico *versus* não-trombolítico. As taxas de mortalidade hospitalar e episódios de sangramento foram definidos como desfechos do estudo. Valor de p <0,05 foi considerado como estatisticamente significativo.

**Resultados:**

A mortalidade hospitalar reduziu significativamente no grupo trombolítico em comparação ao não-trombolítico (10,5% vs. 24,2%; p=0,03). Episódios de sangramento menores foram mais comuns no braço que recebeu o tratamento trombolítico, mas grandes hemorragias não diferiram entre os grupos (35,1% vs. 13,2%, p<0,01; 7% vs. 5,5% p=0,71, respectivamente). O escore de PESI alto (OR: 1,03 IC95%; 1,01-1,04 p<0,01), a terapia trombolítica (OR: 0,15 IC95%; 0,01-0,25, p< 0,01) e níveis altos de troponina (OR: 1,20 IC95%; 1,01-1,43, p=0,03) estiveram independentemente associados a taxas de mortalidade hospitalar na análise de regressão multivariada.

**Conclusão:**

A terapia trombolítica esteve associada à mortalidade hospitalar reduzida em detrimento do aumento geral das complicações de sangramento em octogenários.

## Introdução

A embolia pulmonar é uma das principais causas de morte na população em geral. Sua incidência aumenta com a idade, e pacientes com mais de 65 anos constituem quase 60% desses casos.^1 , 2^ Apesar da alta prevalência de embolia pulmonar entre os idosos, especialmente octogenários, este público costuma estar sub-representado na maioria dos estudos. O manejo deste subgrupo de pacientes ainda não é claro. A presença de doenças cardiovasculares e pulmonares subjacentes pode mascarar os sintomas relacionados à embolia pulmonar, levando ao diagnóstico equivocado ou atrasado.^3^ Assim, as taxas de mortalidade podem ser tão altas quanto 29,5% no curto prazo.^4^

A rápida administração da terapia trombolítica melhora a função ventricular direita, previne o desenvolvimento de choque cardiogênico e mantém a perfusão tissular adequada na ausência de contraindicações.^5^ Apesar da existência de benefício real gerado pela terapia trombolítica no tratamento de pacientes com alto risco de embolia pulmonar, seu uso ainda é baixo.^6^ Preocupações com complicações envolvendo sangramentos, principalmente hemorragia intracraniana, e contraindicações são as principais razões para os clínicos evitarem este tratamento que pode salvar vidas.

Pacientes com mais de 80 anos representam um grupo desafiador, não só por dificuldades relacionadas ao diagnóstico, mas também por apresentarem uma alta carga de comorbidades, mais complicações, como sangramentos, e taxas mais altas de mortalidade. Porém, a idade, por si só, não deveria ser motivo para o baixo uso de modalidades de tratamento que têm base em evidência. Neste estudo, tentamos investigar se esta população específica se beneficia da terapia lítica no cenário da embolia pulmonar aguda.

## Métodos

### População do estudo

Verificamos os registros hospitalares entre 2010 e 2017, retrospectivamente, e analisamos um total de 1.380 indivíduos diagnosticados com embolia pulmonar. Cento e setenta pacientes com mais de 80 anos no momento do diagnóstico foram analisados e, após a exclusão de 22 pacientes com dados faltantes, um total de 148 pacientes foram recrutados consecutivamente para este estudo. Características demográficas, parâmetros laboratoriais, estratégias de tratamento, episódios de sangramento e taxas de mortalidade hospitalares foram registrados. Os desfechos primários do estudo foram mortalidade hospitalar e sangramento. O objetivo foi avaliar o efeito agudo da terapia lítica e suas complicações associadas. O protocolo do estudo foi aprovado pelo Comitê de Ética local.

### Definições

A embolia pulmonar foi diagnosticada com base na presença de achados positivos na angiografia pulmonar computadorizada, como defeito de enchimento completo ou parcial nos ramos principal, lobar, segmentar ou subsegmentar da árvore pulmonar. Pacientes de alto risco foram definidos quando apresentavam instabilidade hemodinâmica levando à parada cardíaca, choque obstrutivo ou hipotensão persistente de acordo com as diretrizes da Sociedade Europeia de Cardiologia – Embolia Pulmonar Aguda (Diagnóstico e Manejo).^7^ O choque obstrutivo foi definido quando a pressão sanguínea sistólica era < 90 mmHg, ou quando eram necessários vasopressores para manter a pressão > 90 mmHg, apesar da pressão de enchimento adequada, além da hipoperfusão do órgão final. A hipotensão persistente foi definida como pressão sanguínea <90 mmHg ou uma redução na pressão sanguínea (> 40 mmHg) durante mais do que 15 minutos na ausência de sepse, nova arritmia ou hipovolemia, enquanto a parada cardíaca significa que há necessidade de reanimação cardiopulmonar (RCP). Pacientes hemodinamicamente estáveis foram classificados como risco intermediário quando o Índice de Gravidade da Embolia Pulmonar (PESI) foi classificado como classe III ou mais, assim como na presença de achados de lesão no miocárdio, assim como níveis altos de troponina e/ou disfunção ventricular direita.^7^ O escore PESI original, incluindo onze parâmetros (idade, sexo masculino, câncer, insuficiência cardíaca crônica, doença pulmonar crônica, frequência cardíaca, frequência respiratória, status mental alterado, temperatura, pressão sanguínea sistólica e saturação de oxihemoglobina), foi calculado para cada paciente.^8^ A hipertensão foi definida quando a pressão sanguínea inicial era >140/90 mmHg ou com uso de anti-hipertensivos. A diabetes mellitus foi definida quando o paciente tomava remédios para diabetes ou quando os níveis de glicose em jejum eram acima de 126 mg/dL. O status sobre o ato de fumar foi definido de acordo com consumo atual de tabaco. A insuficiência cardíaca foi definida quando a fração de ejeção (FE) era menor que 40%, com sintomas consistentes e sinais de insuficiência cardíaca. Repouso por mais de três dias antes da aplicação do índice foi definido como imobilidade. Grandes episódios de sangramento foram definidos como sangramento intracraniano, sinais clínicos claros de sangramento, com redução de níveis de hemoglobina (> 5 gr/dL), ou sangramento fatal, levando diretamente à morte, de acordo com o escore TIMI.^9^ Episódios de sangramento que não estavam de acordo com esses critérios foram definidos como complicações de sangramento menores.

### Terapias de anticoagulação e trombolíticas

Todos os pacientes que não apresentaram instabilidade hemodinâmica seguiram para anticoagulação com heparinas de baixo peso molecular (HBPM) assim que o diagnóstico foi confirmado. A HBMP foi administrada de forma subcutânea, com dose de 1 mg/kg duas vezes ou uma vez por dia, de acordo com as funções renais. Pacientes com insuficiência renal grave foram tratados com heparina não fracionada (HNF). A decisão de implementar a terapia lítica foi tomada pelo primeiro médico atendente em nossa instituição. O ativador do plasminogênio tecidual (tPA) foi o agente lítico padrão para pacientes 14 dias após o aparecimento dos sintomas. Iniciou-se com uma dose intravenosa de 100 mg em um período de 2 horas, e foi coadministrado com HNF na unidade de terapia intensiva após questionamentos sobre contraindicações^7^ e após a assinatura de um termo de consentimento esclarecido. Pacientes receberam HBPM devido à sua fácil administração e porque não requer acompanhamento com níveis de Tempo de Tromboplastina Parcial ativado (TTPa) após o curso da terapia lítica. A varfarina sódica foi iniciada como terapia anticoagulante oral para todos os pacientes na primeira hospitalização. A HBPM continuou com o tratamento com varfarina até que o Índice Internacional Normalizado (INR) atingisse níveis terapêuticos.

### Análise laboratorial

Amostras de sangue foram coletadas na emergência, da veia antecubital, de acordo com os protocolos do hospital. Os níveis de glicose sérica, a concentração de hemoglobina, a contagem de plaquetas e os valores de creatinina sérica e D-dímero foram registrados. Os níveis de Troponina I Cardíaca Humana (cTnI) e Peptídeo Natriurético Cerebral (BNP) foram coletados para futura estratificação de risco.

### Ecocardiograma e Tomografia Computadorizada

Todos os pacientes foram submetidos a um ecocardiograma realizado pelo Sistema Vivid-3 (General Electric, Noruega) durante a avaliação inicial. A dilatação ventricular direita (VD) foi definida com dimensão >3,3 cm, medida a partir do nível médio ventricular no corte apical de quatro câmaras.^10^ A pressão sistólica da artéria pulmonar foi calculada ao adicionar a pressão média do átrio direito ao gradiente de pressão tricúspide, adquirida a partir da velocidade máxima do refluxo tricúspide. A pressão média do átrio direito foi estimada a partir do diâmetro da veia cava inferior e distensibilidade durante o ato de respirar. A pressão média do átrio direito foi considerada como 5 mmHg quando havia colapso completo do vaso e diâmetro normal. Estima-se que há 10 mmHg na presença de >50% de colapso e diâmetro normal. Se o cenário fosse <50% de colapso com a veia cava inferior dilatada, 15 mmHg eram adicionados ao gradiente de pressão tricúspide, e 20 mmHg eram adicionados ao vaso dilatado sem colapsibilidade.^10^ O diagnóstico foi alcançado por meio da angiografia pulmonar por CT espiral, realizada na clínica de radiografia e utilizando o protocolo de embolia pulmonar (campo de visão: 35 cm; espessura da seção: 3 mm; volume do material de contraste: 135 ml; taxa de injeção do contraste: 4 mL/seg), e o exame foi realizado por radiologistas certificados da equipe do hospital. Defeito parcial ou completo no enchimento em um ou mais artérias pulmonares (principal, lobar, segmentar ou subsegmentar) confirmaram o diagnóstico.

### Análise estatística

Todos os dados são apresentados como média ± desvio padrão (DP), para variáveis com distribuição normal, ou mediana [intervalo interquartil] para variáveis com distribuição anormal. As variáveis categóricas são registradas em números ou taxas percentuais. As variáveis contínuas foram verificadas para distribuição normal utilizando o teste de Kolmogorov-Smirnov. As variáveis categóricas foram testadas com o teste qui-quadrado de Pearson e o teste exato de Fisher. As diferenças entre os grupos foram avaliadas com o teste U de Mann Whitney ou o teste t de Student não pareado, quando apropriado. As análises de regressão logísticas binárias univariadas e multivariadas foram realizadas para investigar as correlações independentes da mortalidade hospitalar. Como resultado das análises de regressão univariadas, as variáveis cujos valor de p eram <0,10 foram incluídas nas análises de regressão multivariada. O valor de p era bilateral, e valores <0,05 foram considerados como estatisticamente significativos. Todos os estudos estatísticos foram realizados com o software Statistical Package for Social Sciences (SPSS 22.0 para Windows, SPSS Inc., Chicago, Illinois).

## Resultados

Foram 148 octogenários diagnosticados com embolia pulmonar aguda, e 88,5% (n=131) estavam nas categorias de risco alta ou intermediária. Cinquenta e sete (38,5%) indivíduos do grupo total iniciaram a terapia trombolítica. Nesta população, 28 pacientes (19%) morreram enquanto estavam internados. Enquanto a frequência de sangramentos menores foi de 21,6%, a frequência de grandes sangramentos foi de 6,1%. A idade média da população do estudo foi 83,2±2,9 anos. Além disso, 30% da população do estudo era do sexo masculino.

Características clínicas, demográficas e laboratoriais estão demonstradas nas Tabelas 1 e 2. A idade média foi similar entre os grupos. Os grupos foram estatisticamente comparáveis em termos de parâmetros demográficos e clínicos, exceto pela mortalidade hospitalar e pequenos sangramentos. Enquanto a mortalidade hospitalar ficou mais baixa, os pequenos sangramentos foram mais frequentes no grupo trombolítico em comparação ao grupo não-trombolítico. Quando comparado com o grupo não-trombolítico, o choque foi numericamente mais frequente, e o escore PESI médio foi numericamente mais alto no grupo trombolítico, mas as diferenças não foram estatisticamente significativas. A proporção de pacientes com suporte inotrópico e ventilação mecânica também foram comparáveis entre os grupos.

**Tabela 1:** – Características clínicas e demográficas dos octagenários com embolia pulmonar aguda

	Total (n=148)	Tratamento trombolítıco (n=57)	Tratamento não-trombolítıco (n=91)	p
Idade	83,2±2,9	82,4±2,1	82,6±3,3	0,08
Sexo masculino	44 (29,7%)	16 (28,1%)	28 (30,8)	0,72
Hipertensão	98 (66,2%)	39 (68,4%)	59 (64,8%)	0,72
Diabetes Mellitus	39 (26,4%)	18 (31,6%)	21 (23,1%)	0,25
Fumar	13 (8,8%)	3 (5,3%)	10 (11%)	0,23
ECV	11 (7,4%)	6 (17,5%)	5 (5,5%)	0,25
Insuficiência cardíaca	27 (18,2%)	13 (22,8%)	14 (15,4%)	0,25
Doença pulmonar crônica	25(16,9%)	10 (17,5%)	15 (16,5%)	0,86
Malignidade	22 (14,9%)	9 (15,8%)	13 (14,3%)	0,80
Cirurgia	26 (17,6%)	12 (21,1%)	14 (15,4%)	0,37
Imobilidade	34 (23%)	17 (29,8%)	17 (18,7%)	0,11
Fibrilação atrial	51 (34,5%)	20 (35,1%)	31 (34,1%)	0,89
Dispneia	139 (93,9%)	56 (98,2%)	83 (91,2%)	0,10
Síncope	37 (25%)	21 (36,8%)	16 (17,6%)	0,08
Hemoptise	7 (4,7%)	2 (3,5%)	5 (5,5%)	0,58
Histórico de TVP	9 (6,1%)	2 (3,5%)	7 (7,7%)	0,30
Sinais de TVP	50 (33,8%)	16 (28,1%)	34 (37,4%)	0,24
CHOQUE	46 (31%)	18 (31,5%)	28 (30,7%)	0,1
PESI	126,3±41,2	143,1±36,3	138,6±34,9	0,06
Dimensão do VD (cm)	3,5±0,6	3,9±0,3	3,7±0,6	0,20
PSAP (mmHg)	69,4±18,3	71,2±18,7	67,1±16,8	0,16
Mortalidade hospitalar	28 (18,9%)	6 (10,5%)	22 (24,2%)	0,03
Pequenos sangramentos	32 (21,6%)	20 (35,1%)	12 (13,2%)	<0,01
Grandes sangramentos	9 (6,1%)	4 (7%)	5 (5,5%)	0,71
Ventilação mecânica	15 (10,1%)	6 (10,5%)	9 (9,8%)	0,10
Uso de inotrópicos	36 (24,3%)	13 (22,8%)	23 (25,2%)	0,08

*ECV: Eventos cerebrovasculares; TVP: Trombose venosa profunda; PESI: Índice de Gravidade da Embolia Pulmonar; VD: Ventrículo direito; PSAP: Pressão sistólica da artéria pulmonar.*

**Tabela 2:** – Parâmetros laboratoriais entre os grupos com tratamento trombolítico e não trombolítico

	Total (n=148)	Tratamento trombolítıco (n=57)	Tratamento não-trombolítıco (n=91)	p
CGB (10^3^µ/L)	9,1 [5,1]	11,9 [6,4]	10,8 [4,6]	0,21
Hemoglobina (g/L)	12,5±1,7	12,4±1,7	12,6±1,7	0,44
Plaquetas (/mm^3^)	256,4±101,4	266,7±125,6	250,1±82,9	0,33
Glicose (mg/dl)	142,3±52,5	146,5±57,6	139,7±49,1	0,44
TFG (ml/min/1.73m^2^)	54,6±22,5	53,5±22,2	55,4±22,8	0,62
Sódio (mE/L)	137,5±5,1	137,1±5,3	137,7±5,1	0,48
Potássio (mE/L)	4,2±1,0	4,1±1,2	4,1±0,8	0,99
Troponina I (ng/ml)	0,12 [1]	0,42 [1,3]	0,04 [0,4]	<0,01
BNP (pg/ml)	450 [580]	550 [507]	350 [438]	0,10
D-dímero (ng/ml)	1995 [2927]	2735 [4100]	2040 [2510]	0,20

*CGB: Contagem de glóbulos brancos; TFG: taxa de filtração glomerular; BNP: peptídeo natriurético cerebral.*

Ao olhar para parâmetros laboratoriais, encontramos médias estatisticamente mais altas de troponina I no grupo trombolítico em relação ao não-trombolítico. Os valores médios de BNP e D-dímero também foram mais altos no grupo trombolítico, mas as diferenças não foram estatisticamente significativas.

Na análise de regressão logística univariada, escore PESI, os níveis de troponina e a terapia trombolítica estiveram correlacionados à mortalidade de curto prazo. Quando consideramos essas variáveis na análise de regressão multivariada, determinamos o escore PESI (OR: 1,03 IC95%: 1,01-1,05, p<0,01), a terapia trombolítica (OR: 0,15 IC95%: 0,01-0,25, p<0,01) e a troponina (OR: 1,20 IC95%: 1,01-1,43, p=0,03) como preditores independentes de mortalidade hospitalar ( Tabela 3 ).

**Tabela 3:** – Preditores independentes da mortalidade hospitalar na análise de regressão multivariada

Variável	OR ajustada (IC95%)	p
Escore PESI	1,03 (1,01-1,05)	<0,01
Terapia trombolítica	0,15 (0,01-0,25)	<0,01
Troponina	1,2 (1,01-1,43)	0,03

*PESI: Índice de Gravidade da Embolia Pulmonar.*

De acordo com essas análises de regressão, podemos indicar que pacientes idosos não trombolíticos, com embolia pulmonar aguda, tiveram 6,6 vezes mais riscos de serem acometidos pela mortalidade hospitalar.

Também verificamos a tolerância e o Fator de Inflação da Variância (FIV) para todos os parâmetros incluídos nos modelos para prevenir a multicolinearidade. De acordo com a estatística da multicolinearidade, os valores de tolerância foram >0,1, e os valores de FIV foram <10 para todos os parâmetros. Assim, determinamos que não havia multicolinearidade entre cada uma das variáveis no modelo de regressão.

## Discussão

Os principais achados do nosso estudo são: (1) a terapia trombolítica esteve associada a menores taxas de mortalidade hospitalar entre octogenários; (2) complicações hemorrágicas, de modo geral, foram mais frequentes em pacientes tratados com agentes trombolíticos e primariamente impulsionadas por pequenas hemorragias, sem diferenças em episódios de grandes sangramentos.

A embolia pulmonar é a terceira síndrome cardíaca aguda mais frequente, e é a causa de morte evitável mais comum em pacientes internados.^11^ Pacientes idosos apresentam alta incidência de embolia pulmonar, e também estão mais vulneráveis a complicações, com 2 a 3 vezes mais chances de mortalidade por todas as causas.^2^ Um estudo de corte retrospectivo incluindo 470 pacientes (n=365, idade>65) mostrou taxa de mortalidade geral de 14,2%, que aumentou para 18,9% entre os indivíduos com mais de 80 anos após 30 dias, o que foi comparável ao nosso estudo.^12^ Da mesma forma, um terço dos pacientes com mais de 90 anos morreram devido à embolia pulmonar nos primeiros três meses de tratamento após o diagnóstico no registro RIETE.^13^ Além disso, desfechos adversos relacionados ao tratamento, principalmente complicações envolvendo sangramentos, foram mais frequentemente observados em pacientes idosos. Pacientes com mais de 70 anos têm 4 vezes mais riscos de apresentar grandes hemorragias em comparação a indivíduos mais jovens.^14^

Orientações atuais recomendam a estratificação de risco para decisões de tratamentos em pacientes com embolia pulmonar confirmada.^7^ A embolia pulmonar de alto risco, referida como instabilidade hemodinâmica, foi detectada em uma minoria de pacientes, variando entre 3 e 12% em estudos anteriores.^6 , 15^ Porém, as taxas de mortalidade podem exceder os 50% após 90 dias neste subgrupo de pacientes.^16^ A terapia trombolítica demonstrou reduzir a mortalidade por todas as causas e a mortalidade de curto prazo relacionada à embolia pulmonar em pacientes de risco alto^17^ e intermediário,^18^ independentemente da idade, em detrimento de crescentes episódios de sangramento. Apesar das controvérsias, uma meta-análise revelou que taxas de grandes sangramentos, incluindo hemorragia intracraniana, foram significativamente maiores em pacientes com mais de 65 anos tratados com trombolíticos, em comparação a pacientes que só receberam anticoagulantes (12,9% vs. 4,1%, p<0,001, respectivamente), o que não ficou claro entre pacientes mais jovens.^19^ Por outro lado, Ipek et al. mostrou que pequenas ou grandes complicações de sangramento foram semelhantes entre os grupos que receberam a terapia trombolítica ou não, considerando a análise do subgrupo de pacientes com mais de 65 anos em seu estudo.^20^ Além disso, a mortalidade total foi significativamente menor em pacientes tratados com agentes líticos (7,8% vs. 20,1%, p=0,04). O receio dos médicos em relação a complicações envolvendo sangramentos pode ser uma grande explicação para o baixo uso da terapia trombolítica em pacientes instáveis. Quezada et al.,^21^ demonstrou que somente 23% dos pacientes instáveis receberam terapia trombolítica em sua meta-análise.^21^

Todos os participantes selecionados em nosso corte estavam com risco alto ou intermediário da embolia pulmonar. Em comparação a pessoas mais velhas, a reperfusão foi utilizada de forma mais agressiva (n=57, 38%) em nosso estudo. Ainda assim, deve-se enfatizar que quase dois terços dos pacientes que apresentaram choque não receberam o tratamento trombolítico. A mortalidade hospitalar foi significativamente maior, atingindo 18,9% quando comparada à população geral, provavelmente devido à faixa etária mais velha e à alta porcentagem de pacientes com choque (31%). O escore PESI, a troponina alta e o tratamento de reperfusão foram grandes determinantes da mortalidade hospitalar. A terapia lítica substancialmente reduziu as taxas de mortalidade hospitalar, o que foi compatível com a literatura.^17^ Por outro lado, esta estratégia de tratamento trouxe mais complicações ligadas a sangramentos, como esperado. Pequenos sangramentos foram mais comuns no grupo da terapia trombolítica. Grandes complicações de sangramentos não atingiram significância estatística entre os grupos; porém, isso pode estar relacionado à pequena amostra do estudo. Um paciente teve hemorragia intracraniana e morreu; três outros tiveram sangramento gastrointestinal, que foram manejados com medicamentos.

Em nossa opinião, uma questão se destaca. O ensaio PEITHO, que avaliou principalmente pacientes com risco intermediário de embolia pulmonar, revelou que a terapia trombolítica não estava associada à redução de mortalidade no longo prazo, melhoria da capacidade funcional ou ao desenvolvimento da hipertensão pulmonar tromboembólica crônica.^22^ Porém, deve-se enfatizar que pacientes receberam tenecteplase como o agente lítico neste estudo. Polo Friz et al.,^23^ demonstraram que a sobrevida de longo prazo foi basicamente afetada pelas comorbidades subjacentes, e não pela embolia pulmonar em si, em estudo no qual o número de pacientes com mais de 80 anos era de 46,2%.^23^ Isso significa que octogenários que sobrevivem ao evento agudo e apresentam boas condições de saúde podem se beneficiar mais da terapia trombolítica, e não devem ser considerados como candidatos inadequados para este tratamento por conta de sua idade.

### Limitações do estudo

Nosso estudo tem algumas limitações. Primeiramente, foi um estudo com único centro e retrospectivo, com uma amostra pequena. Benefícios de curto (30 dias) e longo prazo do tratamento lítico não foram avaliados, o que pode influenciar na escolha da estratégia de manejo nesta população específica. Porém, foi difícil avaliar qualquer escore de fragilidade ou contribuição das doenças subjacentes nas taxas de mortalidade, principalmente em pacientes que não receberam a terapia trombolítica. Devido ao pequeno tamanho da amostra e à baixa porcentagem do grupo de risco intermediário e baixo (12%) na população completa do estudo, não pudemos analisar a mortalidade e os episódios de hemorragia separadamente entre esses grupos.

## Conclusão

Nosso estudo demonstrou que a reperfusão reduz a mortalidade hospitalar em octogenários. Embora mais complicações de sangramento tenham ocorrido em indivíduos tratados com trombolíticos, não houve diferenças para grandes hemorragias. Consideramos que esses resultados podem ajudar os médicos a selecionar tratamentos para pacientes idosos.
