# Supplementary material for: Terapia Trombolítica em Octogenários com Embolia Pulmonar Aguda
Source: Arq Bras Cardiol. 2021 Nov 17;118(1):68–74. [Article in Portuguese] doi: 10.36660/abc.20201060 (PMC8959058; doi:10.36660/abc.20201060)
Supplement: Translation

# Translation: Thrombolytic Therapy in Octogenarians with Acute Pulmonary Embolism

**Authors:** Ahmet Zengin, Mehmet Baran Karataş, Yiğit Çanga, Özge Güzelburç, Nizamettin Selçuk Yelgeç, Ayşe Emre

**Affiliations:** 1 Department of Cardiology University of Health Sciences Dr. Siyami Ersek Training and Research Hospital Istambul Turkey Department of Cardiology , University of Health Sciences , Dr. Siyami Ersek Training and Research Hospital , Istambul - Turkey

**Keywords:** Aged, 80 and over, Prognosis, Risk factors, Thrombolytic Therapy, Mortality, Hemorrhage/complications

## Abstract

**Background:**

Despite the high proportion of octogenarians with acute pulmonary embolism, there is little information indicating the optimal management strategy, mainly therapeutic measures, such as lytic therapy.

**Objectives:**

The number of elderly patients diagnosed with acute pulmonary embolism increases constantly. However, the role of thrombolytic treatment is not clearly defined among octogenarians. Our objective is to evaluate the effectiveness of lytic therapy in octogenarian patients diagnosed with pulmonary embolism.

**Methods:**

One hundred and forty eight subjects (70.3% women, n=104) aged more than eighty years were included in the study. The patients were divided in two groups: thrombolytic versus non-thrombolytic treatment. In-hospital mortality rates and bleeding events were defined as study outcomes. P-value <0.05 was considered as statistical significance.

**Results:**

In-hospital mortality decreased significantly in the thrombolytic group compared to the non-thrombolytic group (10.5% vs. 24.2% p=0.03). Minor bleeding events were more common in the arm that received thrombolytic treatment, but major hemorrhage did not differ between the groups (35.1% vs. 13.2%, p<0.01; 7% vs. 5.5% p=0.71, respectively). High PESI score (OR: 1.03 95%CI; 1.01-1.04 p<0.01), thrombolytic therapy (OR: 0.15 95%CI; 0.01-0.25, p< 0.01) and high troponin levels (OR: 1.20 95%CI; 1.01-1.43, p=0.03) were independently associated with in-hospital mortality rates in the multivariate regression analysis.

**Conclusion:**

Thrombolytic therapy was associated with reduced in-hospital mortality at the expense of increased overall bleeding complications in octogenarians.

## Introduction

Pulmonary embolism is a leading cause of death in the general population. Its incidence rises with age, and patients aged more than 65 years constitute nearly 60% of these cases.^1 , 2^ Despite the high prevalence of pulmonary embolism among the elderly, particularly octogenarians, they are frequently underrepresented in most studies. The management of these subgroup of patients are still unclear. The presence of underlying cardiovascular and pulmonary diseases may mask the symptoms related to pulmonary embolism, thus leading to misdiagnosis or delayed diagnosis.^3^ Therefore, mortality rates may be as high as 29.5% in the short term.^4^

The fast administration of thrombolytic therapy improves right ventricular functions, prevents the development of cardiogenic shock and maintains adequate tissue perfusion in the absence of contraindications.^5^ Despite the existence of net clinical benefit due to thrombolytic therapy in the treatment of patients with high risk pulmonary embolism, its use is still low.^6^ Concerns about bleeding complications, particularly intracranial hemorrhage, and contraindications are the main reasons for clinicians to refrain from this life-saving treatment.

Patients aged more than eighty years represent a challenging group, not only for difficulties related to diagnoses, but also because they present a high burden of comorbidities, more complications, such as bleeding, and higher mortality rates. Nevertheless, age alone should not be the reason for the underuse of evidence-based treatment modalities. In this study, we tried to investigate whether or not this highly specific population benefits from lytic therapy in the setting of acute pulmonary embolism.

## Methods

### Study Population

We screened our hospital records between 2010 and 2017, retrospectively, and analyzed a total number of 1,380 subjects diagnosed with pulmonary embolism. One hundred and seventy patients aged more than 80 years at the time of diagnosis were analyzed, and after the exclusion of 22 patients with missing data, a total of 148 subjects were recruited consecutively for this study. Demographic characteristics, laboratory parameters, treatment strategies, bleeding events and in-hospital mortality rates were recorded. The primary outcomes for our study were in-hospital mortality and bleeding. The objective of the study was to evaluate the acute effect of lytic therapy and its associated complications. The study protocol was approved by the local Ethics Committee.

### Definitions

Pulmonary embolism was diagnosed, based on the presence of positive computerized pulmonary angiography findings, as complete or partial filling defect in the main, lobar, segmental or sub-segmental branches of the pulmonary tree. High risk patients were defined as presenting hemodynamic instability encompassing cardiac arrest, obstructive shock or persistent hypotension according to the guidelines from the European Society of Cardiology – Acute Pulmonary Embolism (Diagnosis and Management).^7^ Obstructive shock was defined as systolic blood pressure <90 mmHg or requiring vasopressors to maintain blood pressure >90 mmHg, despite the adequate filling pressures, accompanied by the end organ hypoperfusion. Persistent hypotension was defined as blood pressure <90 mmHg or a reduction in blood pressure (>40 mmHg) lasting more than 15 minutes in the absence of sepsis, new onset arrhythmia or hypovolemia, whereas cardiac arrest means the need for cardiopulmonary resuscitation (CPR). Hemodynamically stable patients were classified as intermediate risk when the Pulmonary Embolism Severity Index (PESI) was class III or higher, and at the presence of findings of myocardial injury, such as elevated cardiac troponin levels and/or right ventricular dysfunction.^7^ The original PESI score including eleven parameters (age, male gender, cancer, chronic heart failure, chronic pulmonary disease, heart rate, respiratory rate, altered mental status, temperature, systolic blood pressure and oxyhemoglobin saturation) was calculated for each patients.^8^ Hypertension was defined as initial blood pressure >140/90 mmHg or using antihypertensives. Diabetes mellitus was defined when the patient was on antidiabetic drugs or when fasting blood glucose levels were above 126 mg/dL. Smoking status was defined as current tobacco use. Heart failure was defined when ejection fraction (EF) was under 40%, with consistent symptoms and signs of heart failure. Bedrest more than three days before the index event was defined as immobility. Major bleeding events were defined as any intracranial bleeding, clear clinical signs of bleeding with a reduction in hemoglobin levels (> 5 gr/dL), or fatal bleeding directly resulting in death according to the TIMI bleeding criteria.^9^ Bleeding events that did not meet these criteria were defined as minor bleeding complications.

### Anticoagulant and Thrombolytic Therapies

All patients without hemodynamic instability anticoagulated with low molecular weight heparin (LMWH) as soon as the diagnosis was confirmed. LMWH was administered subcutaneously dosing 1 mg/kg twice or once a day, according to renal functions. Patients with severe renal failure were treated with unfractionated heparin (UFH). The decision of implementing lytic therapy was left to the discretion of the first attending physician in our institution. Tissue plasminogen activator (tPA) was the standard lytic agent for patients 14 days after the onset of symptoms. It started with an intravenous dose of 100 mg over a 2-hour period, and was co-administrated with UFH in the coronary intensive care unit after questioning for contraindications^7^ and obtaining a written informed consent. Patients received LMWH due to its easy administration and because it does not require follow-up with aPTT levels after the course of lytic therapy. Warfarin sodium was initiated as an oral anticoagulant therapy for all patients at index hospitalization. LMWH continued with warfarin therapy until the international normalization ratio (INR) reached therapeutic levels.

### Laboratory Analysis

Blood samples were taken at the emergency department from the antecubital vein, according to hospital protocols. Admission serum glucose levels, hemoglobin concentration, platelet count, serum creatinine and D-dimer values were recorded. Cardiac troponin I (cTnI) and Brain Natriuretic Peptide (BNP) levels were collected for further risk stratification.

### Echocardiography and Computed Tomography Examinations

All patients underwent an echocardiographic examination performed by Vivid-3 System (General Electric, Norway) during the initial evaluation. Right ventricular (RV) dilatation was defined as RV dimension >3.3 cm, measured from the mid-ventricular level in the apical four chamber views.^10^ Systolic pulmonary artery pressure was calculated by adding the mean right atrial pressure to the tricuspid pressure gradient, which was acquired from the tricuspid regurgitant jet velocity. Mean right atrial pressure was estimated from the inferior vena cava diameter and distensibility while breathing. Mean right atrial pressure was assumed to be 5 mmHg when there was complete collapse of the vessel and normal diameter. It is estimated to be of 10 mmHg in the presence of >50% collapse and normal diameter. If there was <50% collapse with a dilated inferior vena cava, 15 mmHg was added to the tricuspid pressure gradient, and 20 mmHg was added for a dilated vessel without collapsibility.^10^ The diagnosis was achieved by spiral computerized pulmonary angiography, performed in the radiology clinic using the pulmonary embolism protocol (field of view: 35 cm, section thickness: 3 mm, contrast material volume: 135 ml, contrast material injection rate: 4 mL/sec) and examined by certified radiologists from to hospital staff. Partial or complete filing defect in one or more main, lobar, segmental or sub-segmental pulmonary arteries confirmed the diagnosis.

### Statistical analysis

All data are presented as a mean ± standart deviation (SD) for variables with normal distribution, or median [interquartile range] for variables with non-normal distribution. Categorical variables are reported as numbers and percentage rates. Continuous variables were checked for normal distribution using Kolmogorov-Smirnov statistics. Categorical variables were tested by Pearson’s X2 test and Fisher’s Exact Test. Differences between groups were evaluated by using the Mann Whitney U test or the Student t-test as unpaired, when appropriate. Univariate and multivariate binary logistic regression analyses were performed to investigate the independent correlations of in-hospital mortality. As the result of the univariate regression analyses, variables whose p-values were <0.10 were included in the multivariate regression analyses. P-values were two sided, and values < 0.05 were considered as statistically significant. All statistical studies were carried out using the Statistical Package for Social Sciences software (SPSS 22.0 for Windows, SPSS Inc., Chicago, Illinois).

## Results

There were 148 octogenarians diagnosed with acute pulmonary embolism, and 88.5% (n=131) were in the high or intermediate-high risk category. Fifty-seven (38.5%) subjects from the total group began with thrombolytic therapy. In the study population, 28 patients (19%) died during hospitalization. Whereas the frequency of minor bleeding was 21.6%, the frequency of major bleeding was 6.1%. The mean age of the study population was 83.2±2.9 years. Besides, 30% of the study population was from the male gender.

Clinical, demographic, and laboratory characteristics of groups are depicted in Tables 1 and 2. Mean age was similar between groups. The groups were statistically comparable in terms of demographic and clinical parameters, except in-hospital mortality and minor bleeding. While in-hospital mortality was lower, minor bleeding was higher in the thrombolytic group compared to non-thrombolytic group. When compared with the non- thrombolytic group, shock was numerically more frequent, and mean PESI score was numerically high in the thrombolytic group, but the differences were not statistically significant. The proportion of patients with inotropic support and mechanical ventilation were also comparable between groups.

**Table 1:** – Clinical and demographic characteristics of octogenarians presented with acute pulmonary embolism

	Total (n=148)	Thrombolytıc treatment (n=57)	Non-thrombolytıc treatment (n=91)	p
Age	83.2±2.9	82.4±2.1	82.6±3.3	0.08
Male gender	44 (29.7%)	16 (28.1%)	28 (30.8)	0.72
Hypertension	98 (66.2%)	39 (68.4%)	59 (64.8%)	0.72
Diabetes Mellitus	39 (26.4%)	18 (31.6%)	21 (23.1%)	0.25
Smoking	13 (8.8%)	3 (5.3%)	10 (11%)	0.23
CVE	11 (7.4%)	6 (17.5%)	5 (5.5%)	0.25
Heart Failure	27 (18.2%)	13 (22.8%)	14 (15.4%)	0.25
Chronic Pulmonary Disease	25(16.9%)	10 (17.5%)	15 (16.5%)	0.86
Malignancy	22 (14.9%)	9 (15.8%)	13 (14.3%)	0.80
Surgery	26 (17.6%)	12 (21.1%)	14 (15.4%)	0.37
Immobility	34 (23%)	17 (29.8%)	17 (18.7%)	0.11
Atrial Fibrillation	51 (34.5%)	20 (35.1%)	31 (34.1%)	0.89
Dyspnea	139 (93.9%)	56 (98.2%)	83 (91.2%)	0.10
Syncope	37 (25%)	21 (36.8%)	16 (17.6%)	0.08
Hemoptysis	7 (4.7%)	2 (3.5%)	5 (5.5%)	0.58
DVT History	9 (6.1%)	2 (3.5%)	7 (7.7%)	0.30
DVT Signs	50 (33.8%)	16 (28.1%)	34 (37.4%)	0.24
Shock	46 (31%)	18 (31.5%)	28 (30.7%)	0.1
PESI	126.3±41.2	143.1±36.3	138.6±34.9	0.06
RV Dimension (cm)	3.5±0.6	3.9±0.3	3.7±0.6	0.20
sPAP (mmHg)	69.4±18.3	71.2±18.7	67.1±16.8	0.16
In-hospital Mortality	28 (18.9%)	6 (10.5%)	22 (24.2%)	0.03
Minor Bleedings	32 (21.6%)	20 (35.1%)	12 (13.2%)	<0.01
Major Bleedings	9 (6.1%)	4 (7%)	5 (5.5%)	0.71
Mechanical ventilation	15 (10.1%)	6 (10.5%)	9 (9.8%)	0.10
Inotrop use	36 (24.3%)	13 (22.8%)	23 (25.2%)	0.08

*CVE: Cerebrovascular events; DVT: Deep vein thrombosis; PESI: Pulmonary Embolism Severity Index;RV Right ventricle; sPAP: Systolic pulmonary artery pressure.*

**Table 2:** – Laboratory parameters between groups thrombolytic treatment vs non-thrombolytic treatment

	Total (n=148)	Thrombolytıc treatment (n=57)	Non-thrombolytıc treatment (n=91)	p
WBC (10^3^µ/L)	9.1 [5.1]	11.9 [6.4]	10.8 [4.6]	0.21
Hemoglobin (g/L)	12.5±1.7	12.4±1.7	12.6±1.7	0.44
Platelet (/mm^3^)	256.4±101.4	266.7±125.6	250.1±82.9	0.33
Glucose (mg/dl)	142.3±52.5	146.5±57.6	139.7±49.1	0.44
GFR (ml/min/1.73m^2^)	54.6±22.5	53.5±22.2	55.4±22.8	0.62
Sodium (mE/L)	137.5±5.1	137.1±5.3	137.7±5.1	0.48
Potassium (mE/L)	4.2±1.0	4.1±1.2	4.1±0.8	0.99
Troponin I (ng/ml)	0.12 [1]	0.42 [1.3]	0.04 [0.4]	<0.01
BNP (pg/ml)	450 [580]	550 [507]	350 [438]	0.10
D-Dimer (ng/ml)	1995 [2927]	2735 [4100]	2040 [2510]	0.20

*WBC: White blood cell; GFR Glomerular filtration rate; BNP Brain natriuretic peptide.*

When we looked at laboratory parameters, we statistically found higher mean troponin-I levels in the thrombolytic group against the non-thrombolytic group. The mean BNP and D-dimer values were also higher in the thrombolytic group, but the differences were not statistically significant.

In the univariate logistic regression analysis, PESI score, troponin levels and thrombolytic therapy were found to be correlated with short-term mortality. When we consider these variables in the multivariate regression analysis, we determined the PESI score (OR: 1.03 95%CI: 1.01-1.05, p<0.01), thrombolytic therapy (OR: 0.15 95%CI: 0.01-0.25, p<0.01), troponin (OR: 1.20 95%CI: 1.01-1.43, p=0.03) as independent predictors of in-hospital mortality (  Table 3  ).

**Table 3:** – Independent predictors of in-hospital mortality in multivariate regression analysis

Variable	Adjusted OR (95%CI)	p
PESI score	1.03 (1.01-1.05)	<0.01
Thrombolytic therapy	0.15 (0.01-0.25)	<0.01
Troponin	1.2 (1.01-1.43)	0.03

*PESI: Pulmonary Embolism Severity Index.*

According to these regression analyses, we can indicate that non-thrombolytic elderly patients with acute pulmonary embolism had a 6.6-fold increased risk for in-hospital mortality.

We also checked the Tolerance and Variance Inflation Factor (VIF) for all parameters included in the models to prevent multicollinearity. According to the multicollinearity statistic, the tolerance values were >0.1, and VIF values were <10 for all parameters. Therefore, we determined there was no multicollinearity between each of the variables in the regression model.

## Discussion

The main findings of our study are as follows: (1) thrombolytic therapy was associated with lower in-hospital mortality rates among octogenarians; (2) overall hemorrhagic complications were more frequent in patients treated with thrombolytic agents primarily driven by minor hemorrhage, with no differences in major bleeding events.

Pulmonary embolism is the third most frequent acute cardiovascular syndrome and is the most common preventable cause of death in hospitalized patients.^11^ Elderly patients have higher incidence of pulmonary embolism, and they are also more prone to complications, with 2-3 fold increase in all-cause mortality.^2^ A retrospective cohort study including 470 patients (n=365 age>65) showed overall mortality rate of 14.2%, which increased to 18.9% among subjects aged more than 80 after 30 days, which was comparable with our study.^12^ Similarly, one third of the patients aged more than 90 years died due to pulmonary embolism in the first three months of therapy after diagnosis in the RIETE registry.^13^ In addition, treatment-related adverse outcomes, mainly bleeding complications, were more frequently observed in elderly patients. Patients aged more than 70 years have 4 times more risks of presenting major hemorrhage compared with younger subjects.^14^

Current guidelines recommend risk stratification for treatment decisions in patients with confirmed pulmonary embolism.^7^ High risk pulmonary embolism referred as hemodynamic instability was detected in a minority of patients, ranging between 3-12% in previous studies.^6 , 15^ However, mortality rates may exceed 50% after 90 days in this subgroup of patients.^16^ Thormbolytic therapy has been shown to reduce all-cause and pulmonary embolism-related short-term mortality in high^17^ and intermediate risk patients,^18^ regardless of age, at the cost of increased bleeding events. Despite controversies, a meta-analysis revealed that major bleeding rates including intracranial hemorrhage were significantly higher in patients aged more than 65 years treated with thrombolytics compared with patients who only received anticoagulants (12.9% vs. 4.1%, p<0.001, respectively), which was not clear among younger patients.^19^ On the other hand, Ipek et al. showed that minor and major bleeding complications were similar between groups who received thrombolytic therapy or not in the subgroup analysis of patients aged more than 65 years in their study.^20^ Furthermore, total mortality was significantly lower in patients treated with lytic agents (7.8% vs. 20.1%, p=0.04). The fear of physicians about bleeding complications may be the potential explanation for the underuse of thrombolytic therapy in unstable patients. Quezada et al.^21^ demonstrated that only 23% of unstable patients received thrombolytic therapy in their meta-analysis.^21^

All participants recruited in our cohort were at high or intermediate risk of pulmonary embolism. Compared to older ones, reperfusion was used more aggressively (n=57, 38%) in our study. Even so, it should be emphasized that almost two thirds of the patients who presented with shock did not receive thrombolytic therapy. In-hospital mortality was significantly higher, reaching 18.9% when compared with the general population, probably due to older age and the high percentage of patients presenting with shock (31%). PESI score, high troponin and receiving reperfusion treatment were major determinants of in-hospital mortality. Lytic therapy substantially decreased in-hospital mortality rates, which was compatible with the literature.^17^ On the other hand, this treatment strategy came with the results of increased bleeding complications, as expected. Minor bleedings were more frequent in the thrombolytic therapy group. Major bleeding complications did not reach statistical significance between groups; however, this may be owed to the small sample size of the study cohort. One patient suffered from intracranial hemorrhage and died; three other patients had gastrointestinal bleeding, which were medically managed.

In our opinion, one special issue stands out. The PEITHO trial, which predominantly evaluated patients at intermediate risk of pulmonary embolism, revealed that thrombolytic therapy was not associated with long-term reduction of mortality, improvement in functional capacity or development of chronic thromboembolic pulmonary hypertension.^22^ However, it should be emphasized that patients received tenecteplase as the lytic agent in this study. Polo Friz et al.^23^ demonstrated that long-term survival was basically affected by underlying comorbidities rather than pulmonary embolism itself, in a study in which the number of patients aged >80 was 46.2%.^23^ This means that octogenarians who survive the acute event and present good health conditions may benefit most from thrombolytic therapy, and should not be considered as an inappropriate candidate for this treatment because of their age.

### Study limitations

Our study has some limitations. First, it was a single centered and retrospective study design with a small sample. Short (30 days) and long-term benefits of lytic treatment were not assessed, which may influence the choice of management strategy in this specific population. On the other hand, it was hard to evaluate any fragility score or contribution of underlying diseases to mortality rates, particularly in patients who did not receive thrombolytic therapy. Due to the small sample size of our study and the low percentage of the low-intermediate risk group (12%) in the entire study population, we were not able to analyze mortality and hemorrhagic events separately between those groups.

## Conclusıon

In conclusion, our study demonstrated that reperfusion reduces in-hospital mortality in octogenarians. Even though more bleeding complications occurred in subjects treated with thrombolytics, with major hemorrhage it was not different. We think that these results may help clinicians with treatment selection for elderly patients.
